# Supplementary material for: Biomarker Discovery in Rare Malignancies: Development of a miRNA Signature for RDEB-cSCC
Source: Cancers (Basel). 2023 Jun 22;15(13):3286. doi: 10.3390/cancers15133286 (PMC10340387; doi:10.3390/cancers15133286)
Supplement: Supplementary file 1 [file cancers-15-03286-s001.zip › cancers-2359725-supplementary.pdf]

Supplementary Materials

Biomarker discovery in rare malignancies: development of a miRNA signature for RDEB-cSCC

Roland Zauner[1*], Monika Wimmer[1*], Sabine Atzmueller[2], Johannes Proell[2], Norbert Niklas[3], Michael Ablinger[1], Manuela Reisenberger[4], Thomas Lettner[1], Julia Illmer[1], Sonja Dorfer[1], Ulrich Koller[1], Christina Guttmann-Gruber[1], Josefina Piñon Hofbauer[1], Johann W. Bauer[1,4], Verena Wally[1]

[1] EB House Austria, Research Program for Molecular Therapy of Genodermatoses, Department of Dermatology & Allergology, University Hospital of the Paracelsus Medical University, 5020 Salzburg, Austria.
[2] Center for Medical Research, Medical Faculty, Johannes-Kepler-University, 4020 Linz, Austria.
[3] Red Cross Transfusion Service of Upper Austria, 4020 Linz, Austria.
[4] Department of Dermatology & Allergology, University Hospital of the Paracelsus Medical University, 5020 Salzburg, Austria.

* equally contributing authors

Corresponding author:
Roland Zauner, PhD, Phone: +43 57255 82400, e-mail: rolan.zauner@salk.at.
Verena Wally, PhD, Phone: +43 57255 82400, e-mail: v.wally@salk.at

Supplementary Tables

Table S1: Characteristics of HN-SCC balanced training set (balanced) vs tumor cluster I (cluster I) samples.
Table S2: Characteristics of HN-SCC tumor cluster I and II.
Table S3: Coefficients ($ß_{model}$) of glmnet model trained to predict HN-SCC vs RDEB tumor samples.
Table S4: Comparison of performance in predicting RDEB-cSCC tumor samples when trained with different TCGA datasets.
Table S5: Comparison of performance in predicting RDEB-cSCC tumor samples when considering prior knowledge derived from miRNA-target gene expression enrichment analysis.
Table S6: Performance benchmark of SIG-3 model in predicting RDEB tumor samples.
Table S7: Performance benchmark of SIG-33 model in predicting HN-SCC tumor samples.

Supplementary Figures

Figure S1: Expression of HPV infection status-related miRNAs promotes clustering of HN-SCC samples.
Figure S2: Inclusion (CD9) and exclusion markers (GM130) confirming presence of exosomes when interrogating whole cell and exosome protein lysates.
Figure S3: Mature arm selection for TaqMan qPCR validation of up-regulated SIG-10 miRNAs.
Figure S4: Mature arm selection for TaqMan qPCR validation of down-regulated SIG-10 miRNAs.
Figure S5: qPCR validation of SIG-10 miRNAs in RDEB tumor cells.
Figure S6: Evaluation of alternative feature filtering based on prior knowledge.
Figure S7: Using sample weights derived from distance analysis between miRNA expression profiles of RDEB and HN-SCC samples.

Table S1: Characteristics of HN-SCC balanced training set (balanced) vs tumor cluster I (cluster I) samples.

		cluster I	balanced	p-value
**Clinical characteristics**	***Age at initial diagnosis,*** mean ± SD, years	60.0 ± 12.2	61.9 ± 12.4	0.495
	Sex			1.000
	Female	67	6	
	Male	188	16	
	Anatomic site			0.421
	Larynx	50	7	
	Oral cavity	139	10	
	Pharynx	66	5	
	Pathologic T Stage			0.426
	T0	1	0	
	T1	20	4	
	T2	75	7	
	T3	52	3	
	T4	73	5	
	TX	21	2	
	Not reported	13	1	
	Pathologic N Stage			0.377
	N0	82	6	
	N1	30	5	
	N2	88	5	
	N3	3	0	
	NX	38	5	
	Not reported	14	1	
**Risk factors**	***Smoking***			0.559
	Lifelong non-smoker	60	4	
	Current smoker	81	8	
	Reformed smoker (≤15 yrs)	67	4	
	Reformed smoker (>15 yrs)	39	6	
	Reformed smoder (n.a. yrs)	2	0	
	Not reported	6	0	
	Alcohol Consumption			0.819
	No	86	8	
	Yes	166	14	
	Not reported	3	0	
	HPV infection status			1.000
	HPV-	108	11	
	HPV+	17	1	
	Not reported	130	10	
**Molecular subtypes**	***Keratinization***			0.762
	No	215	18	
	Yes	40	4	
	Gene expression subtype			0.086
	Atypical	30	0	
	Classical	24	3	
	Mesenchymal	30	6	
	Basal	41	3	
	Not reported	130	10	

Differences in characteristics between the two datasets were evaluated by two-sample t-test for age, while Fisher's exact tests were applied for all categorical variables. Categories with labels TX, NX and "not reported" were not considered for significance test. Clinical data was retrieved from TCGA. HPV status and molecular subtype was derived from a corresponding dataset published by Lawrence et al [1]. N.a.: not available.

Table S2: Characteristics of HN-SCC tumor cluster I and II.

		Cluster I	Cluster II	p-value
**Clinical characteristics**	***Age at initial diagnosis,*** mean ± SD, years	60.2 ± 12.2	62.1 ± 11.3	0.063
	Sex			0.681
	Female	73	59	
	Male	204	150	
	Anatomic site			0.679
	Larynx	57	49	
	Oral cavity	149	105	
	Pharynx	71	55	
	Pathologic T Stage			0.089
	T0	1	0	
	T1	24	22	
	T2	82	46	
	T3	55	39	
	T4	78	80	
	TX	23	14	
	Not reported	14	8	
	Pathologic N Stage			0.745
	N0	88	75	
	N1	35	31	
	N2	93	64	
	N3	3	3	
	NX	43	27	
	Not reported	15	9	
**Risk factors**	***Smoking***			0.735
	Lifelong non-smoker	64	52	
	Current smoker	89	68	
	Reformed smoker (≤15 yrs)	71	55	
	Reformed smoker (>15 yrs)	45	27	
	Reformed smoder (n.a. yrs)	2	0	
	Not reported	6	7	
	Alcohol Consumption			0.377
	No	94	62	
	Yes	180	142	
	Not reported	3	5	
	HPV infection status			0.554
	HPV-	119	96	
	HPV+	18	11	
	Not reported	140	102	
**Molecular subtypes**	***Keratinization***			0.108
	No	233	187	
	Yes	44	22	
	Gene expression subtype			0.727
	Atypical	30	29	
	Classical	27	18	
	Mesenchymal	36	30	

	Basal	44	30
	Not reported	140	102

Differences in characteristics between the two datasets were evaluated by two-sided t-test for age, while Fisher's exact tests were applied for all categorical variables. Categories with labels TX, NX (main tumor or cancer nearby lymph nodes cannot be measured) and "not reported" were not considered for significance test. Clinical data was retrieved from TCGA. HPV status and molecular subtype was derived from a corresponding dataset published by Lawrence et al [1]. N.a.: not available.

Table S3: Coefficients ($ß_{model}$) of glmnet model trained to predict HN-SCC vs RDEB tumor samples.

miRNA	ßmodel	miRNA	ßmodel	miRNA	ßmodel
hsa-let-7a-1	0.0000	hsa-mir-1910	0.0000	hsa-mir-382	0.0000
hsa-let-7a-2	0.0000	hsa-mir-218-1	0.0000	hsa-mir-411	0.0000
hsa-let-7a-3	0.0000	hsa-mir-218-2	0.0000	hsa-mir-432	0.0000
hsa-let-7b	0.0000	hsa-mir-25	0.0000	hsa-mir-483	0.0000
hsa-let-7c	0.0000	hsa-mir-26a-2	0.0000	hsa-mir-493	0.0000
hsa-let-7d	0.0000	hsa-mir-26b	0.0000	hsa-mir-502	0.0000
hsa-let-7e	0.0000	hsa-mir-296	0.0000	hsa-mir-532	0.0000
hsa-mir-106b	0.0000	hsa-mir-29c	0.0000	hsa-mir-585	0.0000
hsa-mir-1226	0.0000	hsa-mir-301a	0.0000	hsa-mir-589	0.0000
hsa-mir-125b-1	0.0000	hsa-mir-301b	0.0000	hsa-mir-654	0.0000
hsa-mir-125b-2	0.0000	hsa-mir-3176	0.0000	hsa-mir-660	0.0000
hsa-mir-127	0.0000	hsa-mir-337	0.0000	hsa-mir-7-1	0.0000
hsa-mir-1292	0.0000	hsa-mir-339	0.0000	hsa-mir-7-2	0.0000
hsa-mir-1306	0.0000	hsa-mir-33a	0.0000	hsa-mir-7-3	0.0000
hsa-mir-1307	0.0000	hsa-mir-342	0.0000	hsa-mir-744	0.0000
hsa-mir-134	0.0000	hsa-mir-345	0.0000	hsa-mir-760	0.0000
hsa-mir-136	0.0000	hsa-mir-34a	-0.0209	hsa-mir-877	0.0000
hsa-mir-155	0.0000	hsa-mir-3615	0.0000	hsa-mir-9-1	-0.0282
hsa-mir-181a-1	0.0000	hsa-mir-362	0.0000	hsa-mir-9-2	-0.0262
hsa-mir-181a-2	0.0000	hsa-mir-369	0.0000	hsa-mir-9-3	0.0000
hsa-mir-181b-1	0.0000	hsa-mir-370	0.0000	hsa-mir-92b	0.0000
hsa-mir-181b-2	0.0000	hsa-mir-379	0.0000	hsa-mir-95	0.0000

Tumor samples of batch corrected (sva(), removeBatchEffect()) combined HN-SCC cluster I and RDEB cell dataset were used to train a glmnet algorithm with the same hyper-parameter space of lambda and alpha used for training the SIG33 tumor vs normal model. The best performing HN-SCC vs RDEB tumor prediction model running a 5-fold cross-validation chose only n = 3 miRNAs (highlighted in yellow) out of n = 66 listed miRNAs with non-zero coefficients (ßmodel).

Table S4: Comparison of performance in predicting RDEB-cSCC tumor samples when trained with different TCGA datasets.

glmnet model trained on	prediction accuracy (%) in RDEB-cSCC	number of features (miRNAs) used by model
LUAD	100.0	41
LUSC	93.3	6
PRAD	86.7	9
LIHC	86.7	8
HNSC (SIG33)	100.0	33
HNSC (SIG3)	93.3	3

Alternative TCGA miRNA expression datasets (LUAD: Lung adenocarcinoma, LUSC: Lung squamous cell carcinoma , PRAD: Prostate adenocarcinoma, LIHC: Liver hepatocellular carcinoma) were used to train glmnet tumor prediction model. Same procedure including feature pre-filter and 5-fold cross-validation with same hyper-parameter grid-search for optimal lamda and alpha was applied as used for determining SIG33 with HNSC dataset.Table also includes data on full (SIG-33) and sparse (SIG-3) model trained on HNSC cluster I dataset. Normalized and z-transformed miRNA-seq data derived from cultured primary healthy control and RDEB keratinocytes and RDEB-cSCC was used as test dataset for evaluating the prediction performance (accuracy: (TP+TN)/(TP+FP+FN+TN), TP: true positives, TN: true negatives, FP: false positives,FN: false negatives).

Table S5: Comparison of performance in predicting RDEB-cSCC tumor samples when considering prior knowledge derived from miRNA-target gene expression enrichment analysis.

glmnet model trained on	prediction accuracy (%) in RDEB-cSCC	number of features (miRNAs) used by model
HNSC (prior)	93.3	5
HNSC (SIG33)	100.0	33
HNSC (SIG3)	93.3	3

HNSC (cluster I) dataset was used to train glmnet tumor prediction model, although with a pre-filtered miRNA set defined by prior knowledge of putative miRNA-target gene expression modules which where derived from geneset enrichment analysis of cultured primary RDEB-KC/RDEB-cSCC trancriptome data (for details see Fig.S6). Same procedure of 5-fold cross-validation with same hyper-parameter grid-search for optimal lamda and alpha was applied as used for determining SIG33 with HNSC dataset.Table also includes data on full (SIG-33) and sparse (SIG-3) model trained on HNSC cluster I dataset. Normalized and z-transformed miRNA-seq data derived from cultured primary healthy control and RDEB keratinocytes and RDEB-cSCC was used as test dataset for evaluating the prediction performance (accuracy: (TP+TN)/(TP+FP+FN+TN), TP: true positives, TN: true negatives, FP: false positives,FN: false negatives).

Table S6: Performance benchmark of SIG-3 model in predicting RDEB tumor samples.

	Accuracy	ACC 95% CI	Sensitivity	Specificity
RDEB cultured cells	93.33	68.05 - 99.83	83.33	100.00
RDEB exosomes	92.31	64.00 - 99.81	85.71	100.00

Default 0.5 threshold. Accuracy: (true positives + true negatives) / (true positives + false positives + true negatives + false negatives), ACC-CI: accuracy 95 % confidence interval, Sensitivity: true positives / (true positives + false negatives), Specificity: true negatives / (false positives + true negatives)

Table S7: Performance benchmark of SIG-33 model in predicting HN-SCC tumor samples.

	AUC-ROC	Accuracy	Sensitivity	Specificity
HN-SCC clusterI	99.98	73.29	69.06	100.00
HN-SCC clusterII	99.61	67.97	64.59	100.00

Default 0.5 threshold. AUC-ROC: % area under the receiver operating curve. HN-SCC: cluster I entailing test set samples.

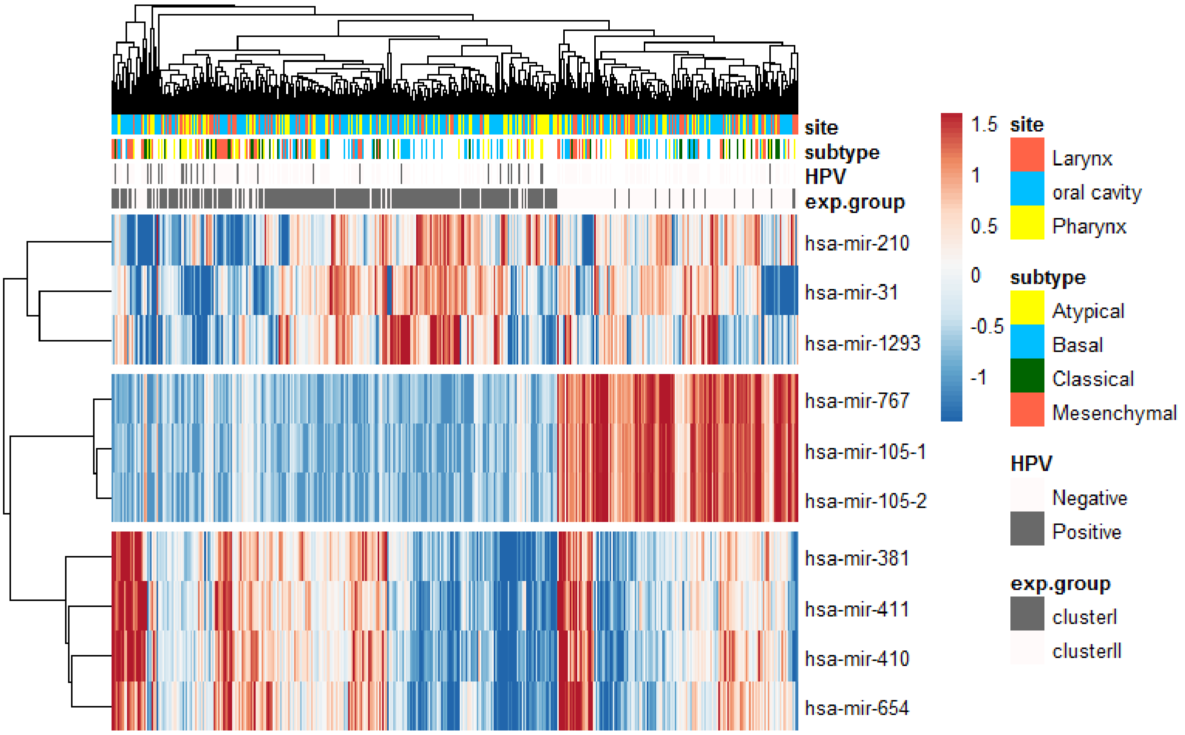

Figure S1: Expression of HPV infection status-related miRNAs promotes clustering of HN-SCC samples. *Heatmap of top ten miRNAs (rows) contributing to PCA Dim2 which drive the separation of HN-SCC clusters I and II (samples in columns). Cluster I samples demonstrate lower expression (blue) of mir-767, mir-105-1 and mir-105-2 compared to cluster II samples, which is associated with positive HPV infection status in cervical SCCs as well as in oropharyngeal carcinoma cells [2,3]. Agglomerative hierarchical clustering was performed in R on euclidean distances with average linkage method, miRNA expression data were centered and scaled. Columns were annotated with curated TCGA clinical data (site: anatomical site) as well as data derived from Lawrence et al (HPV: HPV infections status, subtype: gene expression subtype), exp.group: HN-SCC samples labelled with cluster I and II, according to their separation in PCA Dim2 [1].*

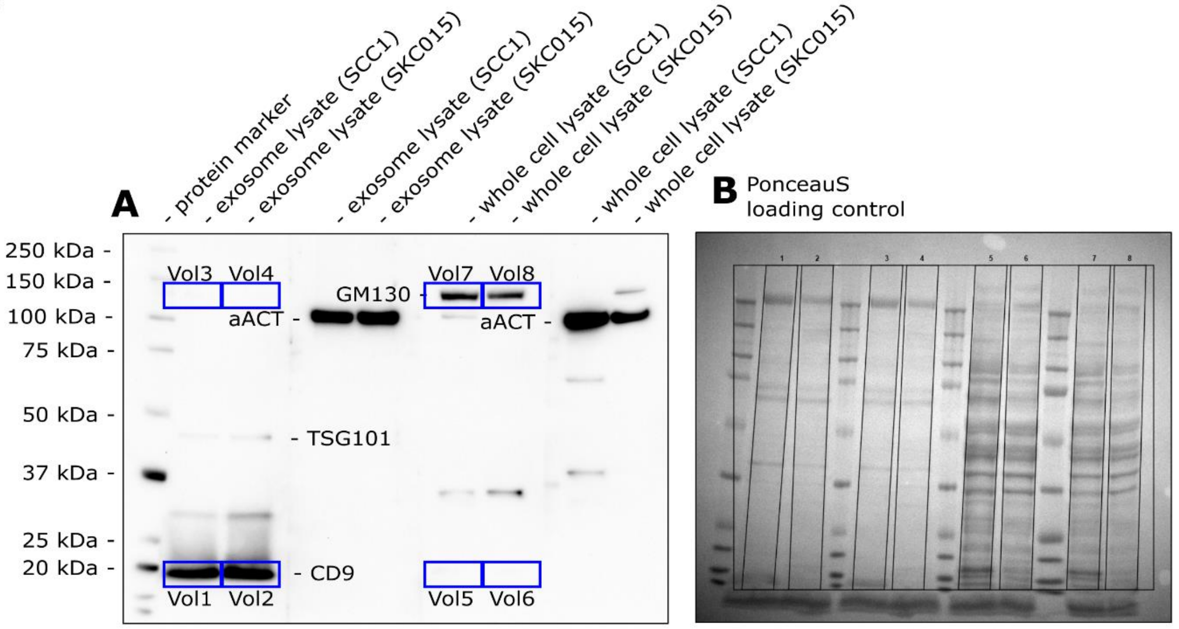

C ***densiometry of WB bands:***

protein band	background adjusted integrated intensity
Vol1	7.180.804
Vol2	9.887.853
Vol3	16.425
Vol4	-6.885

protein band	background adjusted integrated intensity
Vol5	3.307
Vol6	16.685
Vol7	3.322.232
Vol8	2.565.416

Figure S2: Inclusion (CD9) and exclusion markers (GM130) confirming presence of exosomes when interrogating whole cell and exosome protein lysates. *(A) Western blot (WB) with blue boxes indicating the volumetric intensity densiometry of marker bands, molecular weights of protein standards are labelled on the left side (Precision Plus Protein Dual Color Standard, Bio-Rad, Hercules, CA, US), sample type loaded into lanes are labelled on top of the blot, GM130 (1:1000 dilution of cs12480S, Cell Signaling Technology, Cambridge, UK), TSG101 (1:500 dilution of GTX70255, GeneTex, Irvine, CA, US), CD9 (1:100 dilution of sc13118, Santa Cruz, Dallas, US), aACT (1:1000 dilution of sc15335, Santa Cruz, Dallas, US). (B) PonceauS staining showing equal protein loading, image acquired with ChemiDoc imager (Bio-Rad Laboratories, Hercules, CA, US). (C) Densitometry data of marker bands representing HRP activity assessed with Amersham ECL Select Western blot detection reagent (Amersham Biosciences, Little Chalfont, UK) measured in Image Lab software (version 6.0.1, Bio-Rad Laboratories, Hercules, CA, US) by using "volume tools".*

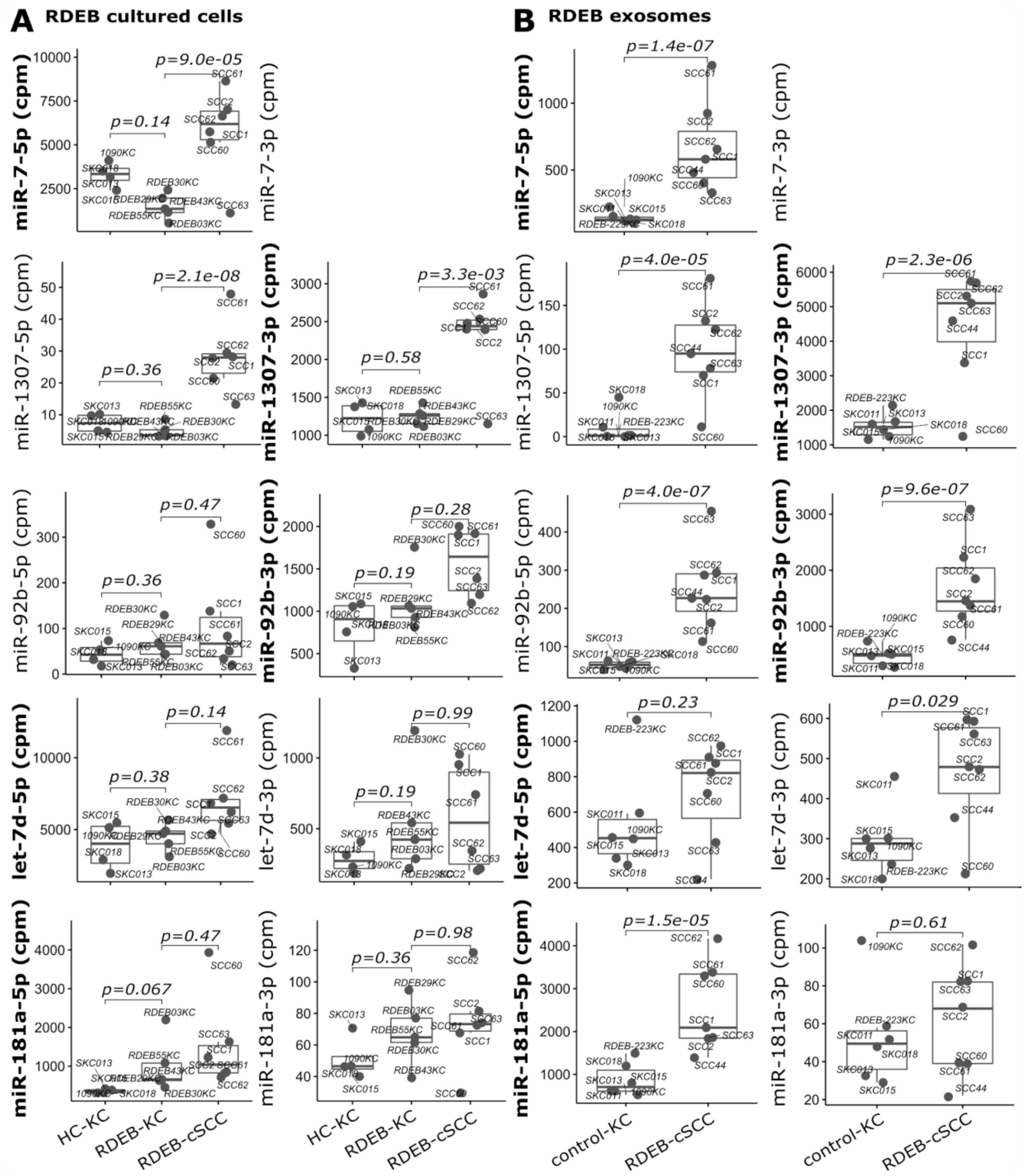

***Figure S3*: Mature arm selection for TaqMan qPCR validation of up-regulated SIG-10 miRNAs.** *Boxplots show miRNA sequencing counts mapped to mature miRNAs (-5p/-3p arm), normalized by library size (mapped reads, cpm: counts per million) of five miRNAs used in the prediction model (p-value: significance, DESeq2 Wald test, Benjamini & Hochberg multiple testing adjusted). Each dot represents one sample, in case of undetectable/too low counts no boxplot is shown for the respective arm. The respective predominant arms, which were used for TaqMan qPCR validation, are highlighted in bold. For exosomes, RDEB- and HC-KCs were grouped as "control-KC".*

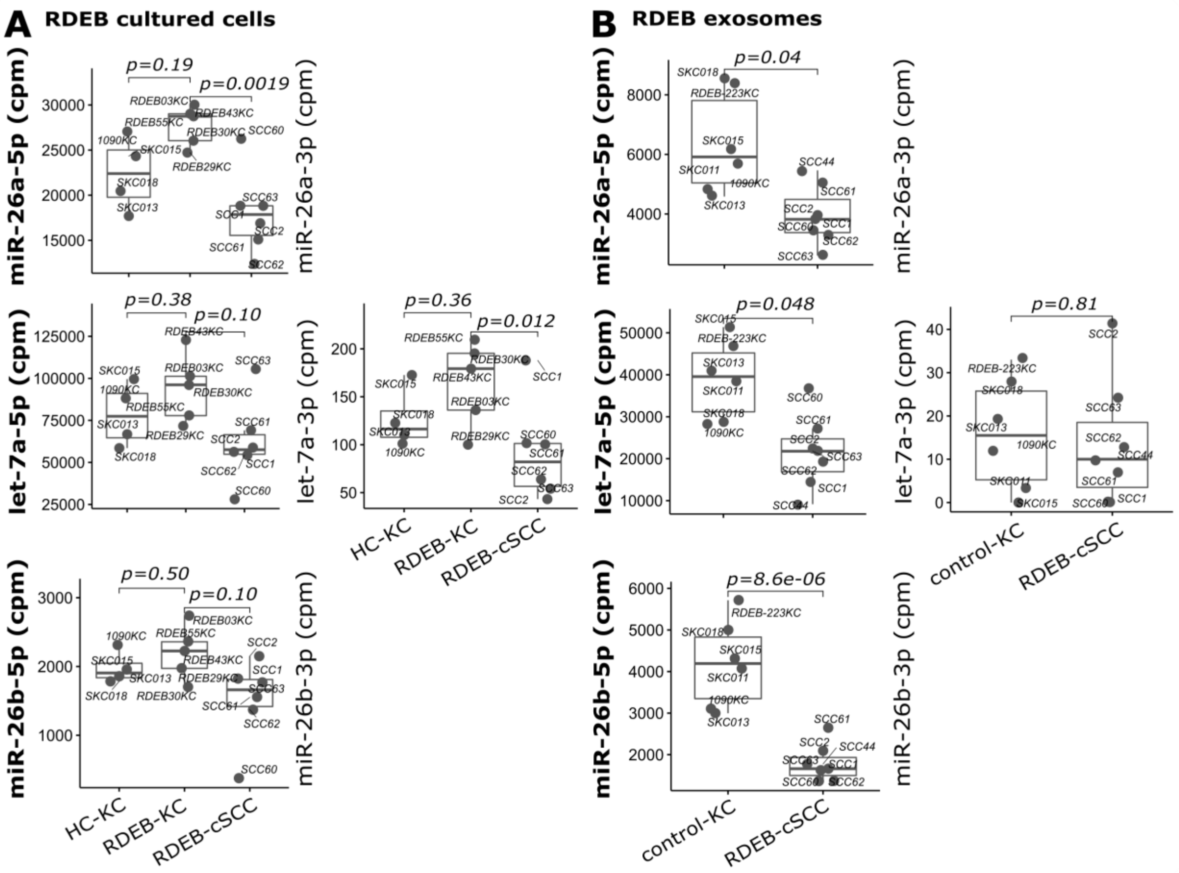

Figure ***S4*****: Mature arm selection for TaqMan qPCR validation of down-regulated SIG-10 miRNAs.** *Boxplots show miRNA sequencing counts mapped to mature miRNAs (-5p/-3p arm), normalized by library size (mapped reads, cpm: counts per million) of three miRNAs used in the prediction model (let-7a-1/2/3 result in only one mature miRNA: let-7a-5p/-3p, p-value: significance, DESeq2 Wald test, Benjamini & Hochberg multiple testing adjusted). Each dot represents one sample, in case of undetectable/too low counts no boxplot is shown for the respective arm. The respective predominant arms, which were used for TaqMan qPCR validation, are highlighted in bold. For exosomes, RDEB- and HC-KCs were grouped as "control-KC".*

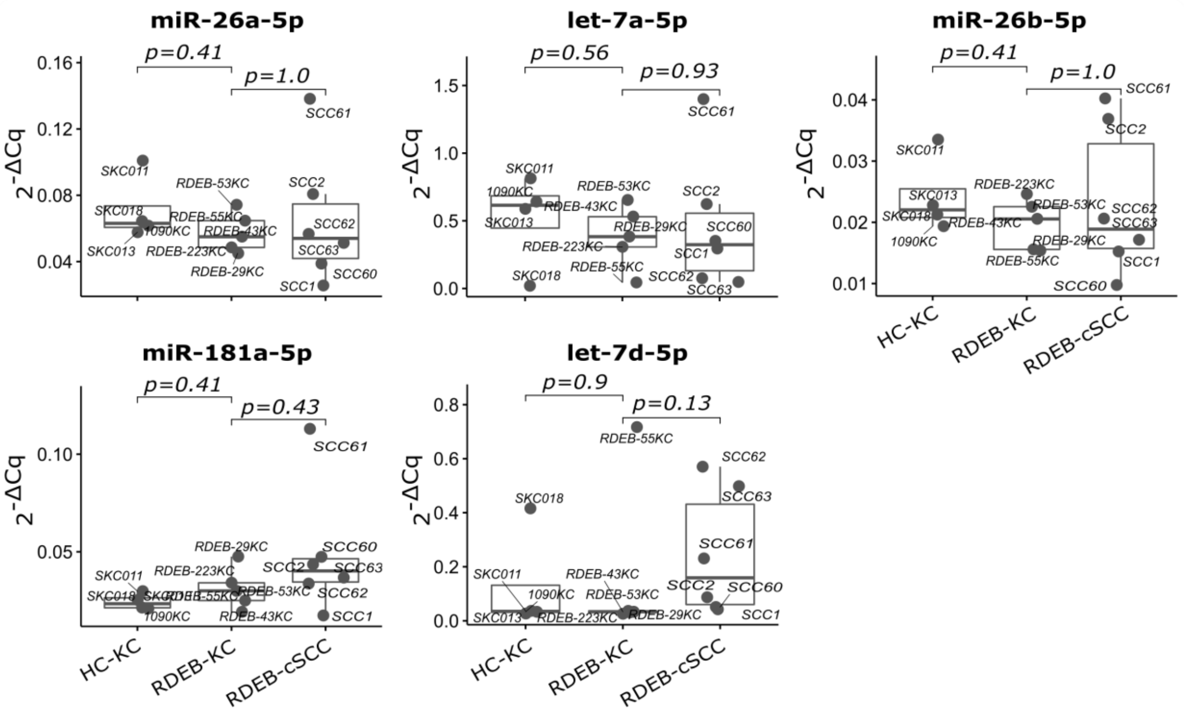

Figure S5: qPCR validation of SIG-10 miRNAs in RDEB tumor cells. *Boxplots show relative expression of five non-significant mature miRNAs derived from the SIG-10 prediction model. TaqMan qPCR was performed on cultured RDEB cells (p-value: significance, non-parametric unpaired Wilcox test, geometric mean of 5S RNA and RNU5G was used as reference). Each dot represents one sample.*

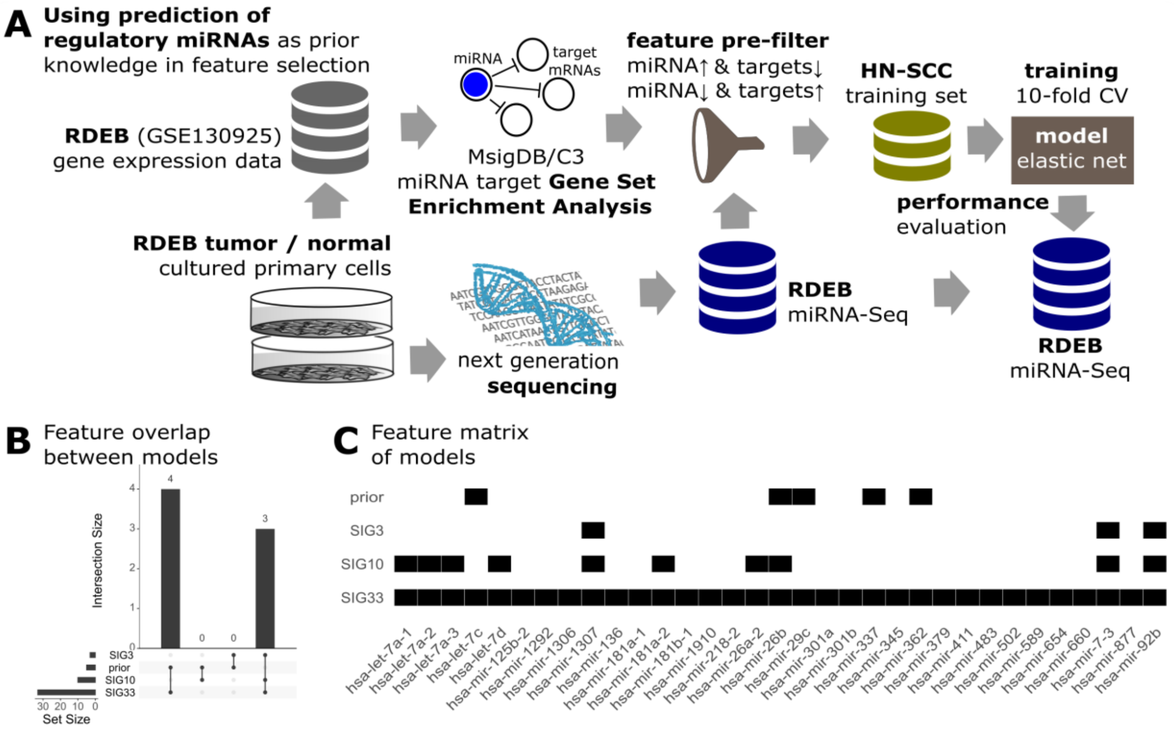

Figure S6: Evaluation of alternative feature filtering based on prior knowledge. *(A) Workflow illustrates the data processing scheme – normalized and log*$_2$ *transformed gene expression data derived from cultured primary RDEB-KC (n = 6) and RDEB-cSCC (n = 4) were retrieved from GEO repository (GSE130925 [4]) used to determine differential gene expression via Bioconductor/limma framework. The t-statistic of the gene expression profiles between RDEB-cSCC and RDEB-KC was used in gene set enrichment analysis of MSigDB C3 miRNA target gene sets applying Bioconductor/fgsea algorithm. A list of upregulated (logFC > 0) miRNAs in RDEB-cSCC (miRNA-Seq dataset) which are associated with significantly enriched (FDR < 0.1) miRNA target genesets indicating downregulation of genes in RDEB-cSCC (RDEB gene expression dataset) and vice versa was considered as prior knowledge about putative miRNA-target gene regulation modules and used as pre-filter to narrow down the features list (miRNAs) used in the HN-SCC training during model training (designated as "prior" model in B,C) applying the same parameters (lamda, alpha) used for deriving the SIG33 signature. The model performance in predicting tumor samples was evaluated using the RDEB miRNA-Seq dataset (cultured primary cells). (B) Upset plot shows number of features/miRNAs (Set Size) used by the models number of features shared between models (Intersection Size). (C) Heatmap indicates features/miRNAs shared between the models (black box: miRNA used in model).*

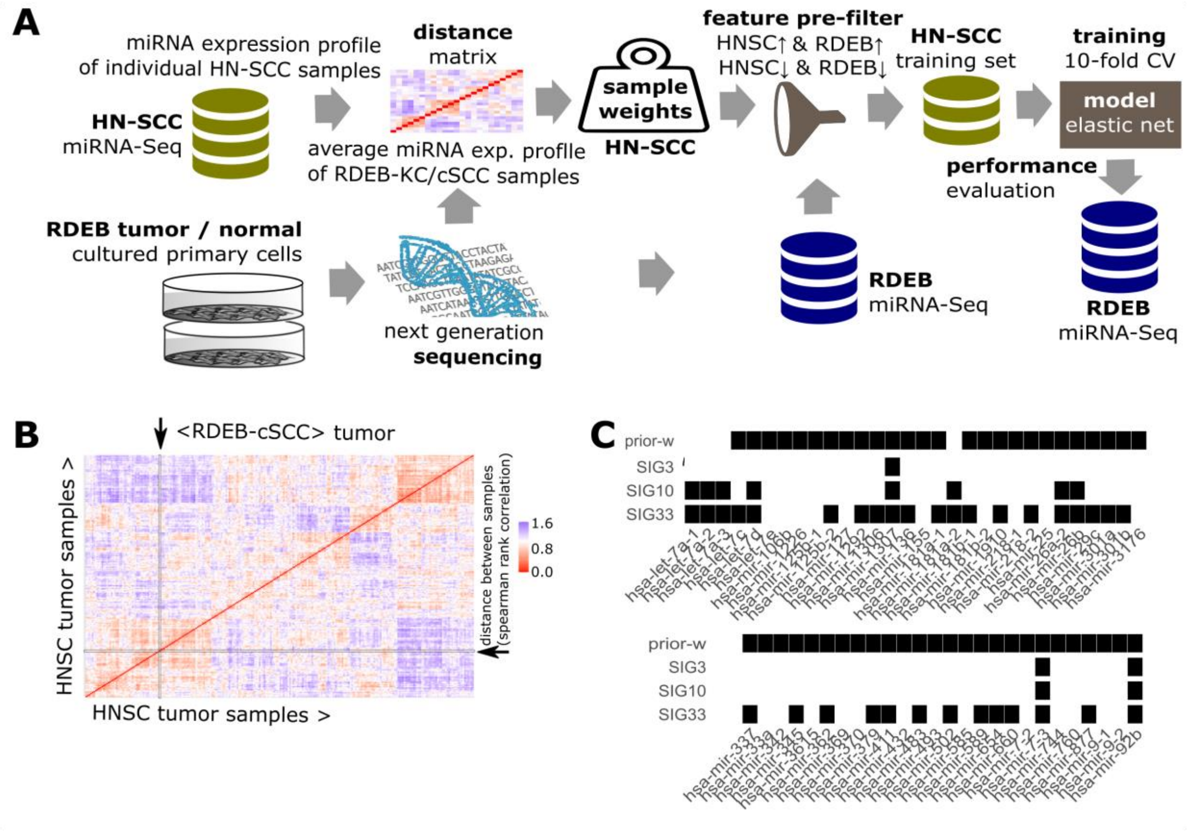

Figure S7: Using sample weights derived from distance analysis between miRNA expression profiles of RDEB and HN-SCC samples. *(A) Workflow outlines the alternative "prior-w" model training procedure. A distance matrix based determined by get_dist function of factoextra R package using spearman rank correlation between mean or normalized miRNA counts across tumor/control RDEB samples and normalized miNRA counts of individual HN-SCC samples was min/max-scaled to derive HN-SCC sample weights. An elastic net model was trained with all (cluster I+II) HN-SCC samples using same feature pre-filter as well as grid-search parameters for optimal alpha/lambda in 5-fold cross-validation as used for SIG-33 model. Sample weights were provided to the glmnet algorithm as additional prior information during model training. The prediction performance (100% accuracy using 52 features/miRNAs) of the "prior-w" model was evaluated using the cultured primary RDEB miRNA-Seq dataset. (B) Heatmap of distances determined from spearman rank correlations comparing miRNA expression profiles of individual HN-SCC samples with mean across RDEB-cSCC tumor samples. Extra matrices were calculated for tumor and normal samples. (C) Plot shows overlap in used features/miRNAs between different models.*

References

1. *Lawrence MS, Sougnez C, Lichtenstein L, Cibulskis K, Lander E, Gabriel SB, et al. Comprehensive genomic characterization of head and neck squamous cell carcinomas. Nature. Nature Publishing Group; 2015;517:576–82.*

2. *Burk RD, Chen Z, Saller C, Tarvin K, Carvalho AL, Scapulatempo-Neto C, et al. Integrated genomic and molecular characterization of cervical cancer. Nature. Nature Publishing Group; 2017;543:378–84.*

3. *Peacock B, Rigby A, Bradford J, Pink R, Hunter K, Lambert D, et al. Extracellular vesicle microRNA cargo is correlated with HPV status in oropharyngeal carcinoma. J Oral Pathol Med. 2018;47:954–63.*

4. *Wimmer M, Zauner R, Ablinger M, Piñón-Hofbauer J, Guttmann-Gruber C, Reisenberger M, et al. A cancer stem cell-like phenotype is associated with miR-10b expression in aggressive squamous cell carcinomas. Cell Commun Signal. 2020;18:61*
